# The intracellular chylomicron highway: novel insights into chylomicron biosynthesis, trafficking, and secretion

**DOI:** 10.1097/MOL.0000000000000983

**Published:** 2025-03-28

**Authors:** Ankia Visser, M. Mahmood Hussain, Jan Albert Kuivenhoven

**Affiliations:** a Department of Pediatrics, University Medical Centre Groningen, University of Groningen, Groningen, The Netherlands; b Department of Foundations of Medicine, NYU Long Island School of Medicine, Mineola, New York, USA

**Keywords:** apoB, chylomicrons, lipoprotein biosynthesis, small intestine

## Abstract

**Purpose of review:**

Chylomicron biosynthesis plays a vital role in supplying essential lipids and lipid soluble vitamins to peripheral tissues for various functions. Despite this, the intracellular synthesis, trafficking, and secretion of chylomicrons remains only partly understood. The purpose of this review is to summarize the role of established proteins in this process and bring attention to recently identified proteins to provide an up-to-date model of chylomicron biosynthesis.

**Recent findings:**

Recently, several proteins have been shown to play a role in the initial formation and lipidation of chylomicrons at the endoplasmic reticulum (ER), which include: TM6SF2, PLA2G12B, PRAP1, and SURF4. In addition, mitochondria have been implicated in chylomicron metabolism, but mechanistic insight is missing. The trafficking of chylomicrons from the ER to the Golgi, and the subsequent trafficking from the Golgi to the basolateral side of enterocytes, however, remains a mystery.

**Summary:**

Progress in the chylomicron biosynthesis field is largely associated with findings in VLDL biosynthesis. In addition, increased insight in events after prechylomicrons leave the ER is needed. Given the important role of chylomicron biosynthesis in whole-body lipid metabolism, further research into the molecular mechanisms is warranted.

## INTRODUCTION

The small intestine is the key organ in dietary lipid absorption. Lipids are broken down in the intestinal lumen and hydrolysed products are absorbed by the enterocytes. Lipids are then re-synthesized and packaged into lipoproteins called chylomicrons. Chylomicrons are secreted into the lymphatics and made available to peripheral tissues after delivery into the circulation *via* the thoracic duct. This process supplies essential fatty acids and lipid soluble vitamins to different tissues for various functions prior to the removal of chylomicron remnants by the liver. The intracellular biosynthesis, trafficking, and secretion of chylomicrons remain incompletely understood despite its vital role in whole-body lipid metabolism. Established factors that regulate assembly and secretion of chylomicrons are described in excellent reviews [1,2]. The purpose of this review is to provide an up-to-date model of chylomicron biosynthesis, transport and secretion. 

**Box 1:**
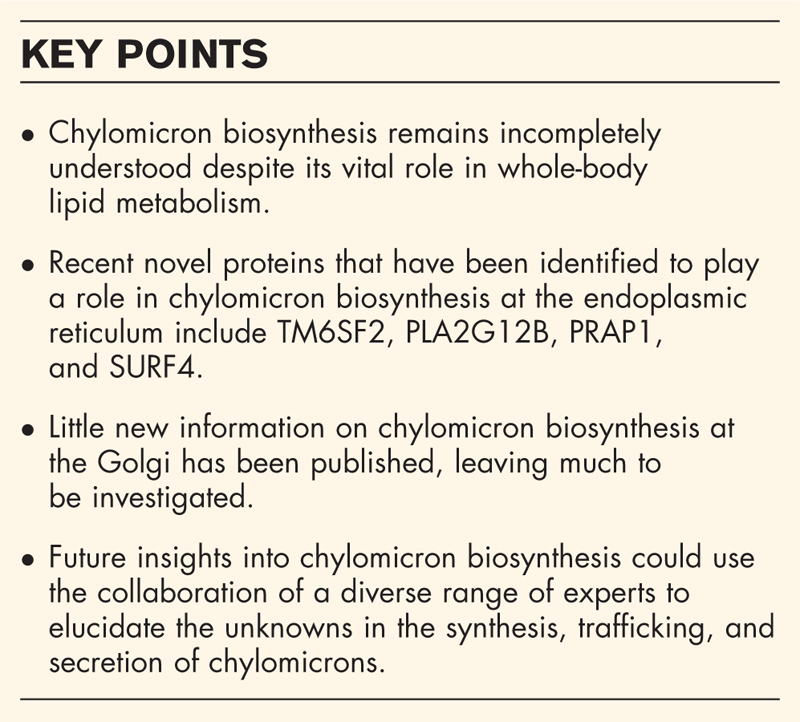
no caption available

## BUILDING BLOCKS FOR CHYLOMICRON BIOSYNTHESIS

The essential proteins for chylomicron assembly are apolipoprotein B48 (apoB48), a scaffolding protein, and microsomal triglyceride transfer protein (MTP) which is needed for apoB48 lipidation. MTP associates with the endoplasmic reticulum (ER) chaperone protein disulfide isomerase (PDI), a subunit of MTP, thereby promoting MTP's lipid transfer activity [3]. The main chylomicron lipid species are triglycerides. Other minor lipids present are phospholipids, free and esterified cholesterol. The lipid cargo of chylomicrons is mainly derived from two sources: dietary lipids and lipids stored in intracellular cytosolic lipid droplets (LDs). Briefly, the precursors required for triglyceride and phospholipid synthesis, fatty acids and monoglycerides, are taken up either passively when the concentration in the intestinal lumen is higher than within the enterocyte, or *via* cluster of differentiation 36 (CD36) and fatty acid transport protein 4 (FATP4) when luminal concentrations are lower. Free cholesterol is primarily taken up by Niemann-Pick C1-like 1 (NPC1L1) but scavenger receptor class B type 1 (SR-B1) is also reported to play a role: SR-BI knockout mice have reduced chylomicron production [4,5]. Lysophospholipids passively diffuse across the enterocytes’ membrane. More detailed information on intestinal lipid absorption can be found elsewhere [6,7].

The fatty acids and monoacylglycerols are first re-esterified in the ER before they are incorporated into the chylomicrons or LDs. Triglycerides are synthesized in the intestine mainly *via* the monoacylglycerol pathway. Briefly, monoacylglycerol acyltransferases initiate the esterification of monoglycerides with fatty acyl-CoA to form diglycerides. Fatty acyl-CoA itself is formed by the action of acyl-CoA synthetases, which esterifies fatty acids. Diacylglycerol acyltransferases, further esterify diglycerides into triglycerides. Cholesterol is esterified by acyl-CoA:cholesterol acyltransferases, and lysophospholipids by acyltransferases. Detailed explanations on how the precursors reach the ER membrane and their subsequent re-esterification can be found in [1,8].

Preexisting cytosolic LDs are hydrolysed, whereby fatty acids are transported to the ER, lipids are re-synthesized, and then incorporated into chylomicrons [9]. Several proteins associated with cytosolic LDs are linked to lipoprotein production through hydrolysis and re-esterification mechanisms that involve amongst others perilipin 2 (PLIN2), perilipin 3 (PLIN3), adipose triglyceride lipase (ATGL), and hormone-sensitive lipase (HSL) [10–13]. However, their role in chylomicron biosynthesis have not been well established.

There is also data showing that an additional lipid source for chylomicrons comes from *de novo* lipogenesis [14], however, *de novo* lipogenesis rates are much lower in the intestine compared to the liver and adipose tissues [14–16].

## THE BIOSYNTHESIS OF THE PRE-CHYLOMICRON IN THE ENDOPLASMIC RETICULUM

**FIGURE 1:**
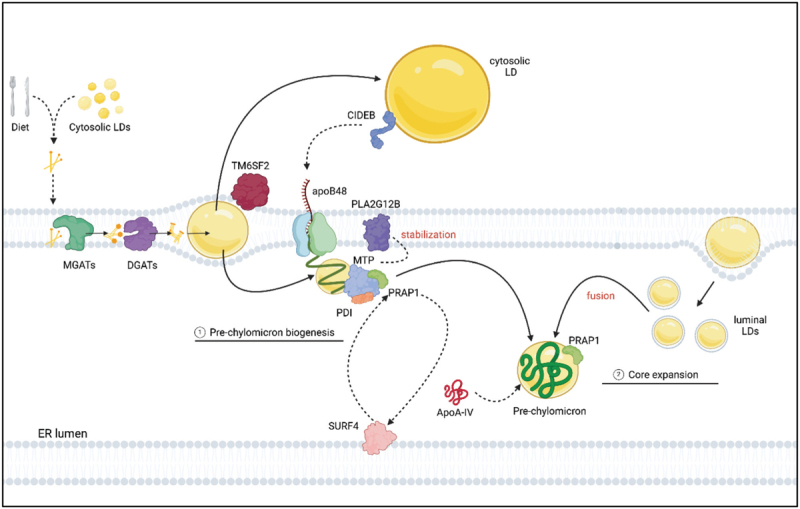
The formation and core expansion of chylomicrons at the endoplasmic reticulum (ER). Fatty acids and monoacylglycerols mainly from the diet and/or hydrolysed cytosolic LDs are re-esterified at the ER by MGATs and DGATs to create a lipid lens within the ER membrane. □ ApoB48 is co-translationally lipidated by MTP; a process that involves subunit PDI and PRAP1. PRAP1 is incorporated into the prechylomicron by SURF4. PLA2G12B has also been shown to aid in the lipidation of chylomicrons by stabilizing MTP to the ER membrane. CIDEB is a LD protein that interacts with apoB48 at the ER membrane. TM6SF2 has also been implicated in the lipidation of chylomicrons but its’ exact role is not known. ApoAIV is incorporated into the chylomicron at the ER and has been shown to play a role in the size expansion of chylomicrons. □ Further lipidation of nascent chylomicrons to prechylomicrons of up to 500 nm is thought to occur *via* fusion to luminal LDs but the mechanisms are not known. Abbreviations: LDs, lipid droplets; MGAT, monoacylglycerol acyltransferase; DGATs, diacylglycerol acyltransferases; apoB48, apolipoprotein B48; MTP, microsomal triglyceride transfer protein; PDI, protein disulfide isomerase; PRAP1, proline-rich acidic protein 1; SURF4, surfeit locus protein 4; PLA2G12B, phospholipase A2 group B12; CIDEB, cell death-inducing DFFA-like effector b; TM6SF2, transmembrane 6 superfamily member 2; apoAIV, apolipoprotein AIV. Figure made in BioRender.

Chylomicron biosynthesis occurs in two stages: the production of a lipid-poor prechylomicron lipoprotein particle, and core expansion through additional lipidation [17] (see Fig. 1). Biosynthesis of chylomicrons starts with the translation of apoB mRNA. In the intestine, the majority of the apoB mRNA transcripts undergo posttranscriptional modifications after which the apoB48 protein is translated. Some studies show that the human small intestine can also synthesize apoB100, although apoB48 is the principal apolipoprotein present [18,19]. Regardless, apoB undergoes co-translational lipidation [20]. In the human hepatocytes, an inadequate lipid supply to apoB100 results in ER-associated degradation [21] but it is unclear if this also occurs to poorly lipidated apoB48 in enterocytes. While germline ablation of either *Apob* or *Mttp* genes in mice results in embryonic lethality [22–24], intestine-specific *Mttp* deletion in mice are viable and show a significant reduction in circulating apoB48 [25].

In recent years, there have been several proteins described that play a role in the initial formation and lipidation of the chylomicron which we wish to highlight.

Transmembrane 6 Superfamily Member 2 (TM6SF2) was initially implicated in the lipidation of nascent apoB-containing lipoproteins in the ER in human and mouse liver [26,27]. Suggestive of a similar function of TM6SF2 in the intestine, O’Hare *et al.* showed that depletion of TM6SF2 in Caco-2 also results in intracellular lipid accumulation while loss of *tm6sf2* in zebrafish induced the accumulation of triglycerides and decreased lipid clearance in the liver as well as the small intestine [28].

Cell death-inducing DFFA-like effector b (CIDEB) is a LD protein with the ability to interact with apoB48 in Caco-2 cells [29]. A physiological role for CIDEB is highlighted with *Cideb*-deficient mice displaying a reduction in chylomicron-triglycerides in the circulation, with a marked lipid accumulation in the enterocytes [29]. CIDEC, is also implicated in intestinal lipid homeostasis, as demonstrated in an intestine-specific *Cidec* knockout mouse model [30]. On a high-fat diet, these mice display reduced body weight gain, fat mass, and lean mass in a context of reduced energy expenditure. In addition, they are characterized by reduced circulating plasma triglycerides and higher faecal triglycerides [30], but whether it has a specific role in chylomicron production is not described.

Recently, phospholipase A2 group B12 (PLA2G12B), a catalytically inactive lipase that has been identified as another key role-player in apoB-lipoprotein assembly in zebrafish larvae and mouse liver [31^▪^]. It is proposed to concentrate and stabilize MTP to the ER membrane, thereby enhancing the lipidation of apoB. *Pla2g12b*^*bsa659*^ mutant zebrafish display hepatic as well as intestinal lipid accumulation [31^▪^]. Furthermore, loss of PLA2G12B in a human intestinal cell line (Caco-2) resulted in lipid-poor lipoprotein-like particles being secreted.

Proline-rich acidic protein 1 (PRAP1), abundantly expressed in the small intestine of humans and mice, plays a role in promoting the lipid transfer activity of MTP by forming a ternary complex with triglycerides and MTP in the ER. PRAP1 deficient mice secrete less apoB, smaller lipoproteins, gain less weight, and accumulate lipids in the small intestine [32^▪^]. PRAP1 is found to be incorporated into the chylomicron by Surfeit locus protein 4 (SURF4), a protein cargo receptor in the ER. Loss of *Surf4* in the intestine of mice causes accumulation of PRAP1 in the small intestine along with a reduction in PRAP1, triglycerides, apoB48 but also apoAI in the plasma [33].

Furthermore, the lipid-poor chylomicron has been proposed to fuse to small lipid droplets in the lumen of the ER [1,2,13,34]. Given an adequate lipid supply and optimal functioning of the above-mentioned proteins, it is assumed that the outcome of the first stage in chylomicron biosynthesis is a lipid-poor chylomicron particle.

While numerous apolipoproteins have been identified on chylomicrons isolated from the circulation [35,36], contradictory roles for apolipoprotein AIV (apoAIV) in chylomicron assembly have been proposed. However, ApoAIV is incorporated into the primordial chylomicron at the ER where it impacts chylomicron size [37,38].

## THE FORMATION AND TRAFFICKING OF PCTVS

The conclusion of chylomicron expansion in the ER is followed by preparation for the exit from the ER in so-called prechylomicron transport vesicles (PCTVs) [39]. The importance of chylomicron transport from the ER was recognized upon the discovery of chylomicron retention disease (OMIM #246700) due to mutations in secretion associated Ras related GTPase 1B (SAR1B*)*[40–42]. In this disease, chylomicrons are formed but they fail to secrete.

**FIGURE 2:**
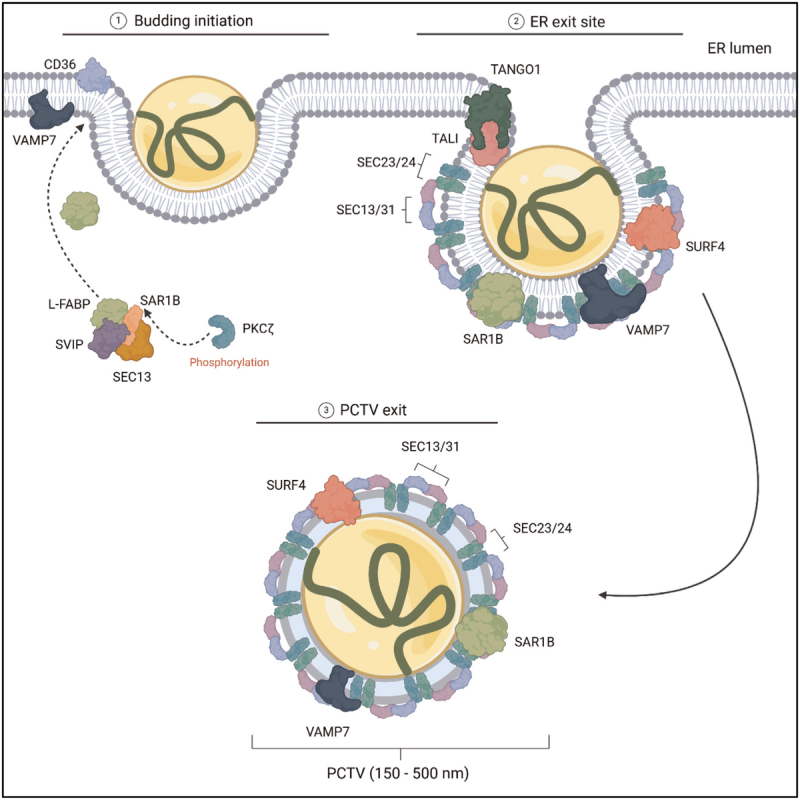
Proteins involved in prechylomicron transport vesicle (PCTV) budding and subsequent exit from the endoplasmic reticulum (ER). □ The SAR1B, SVIP, and SEC13 protein complex inhibits L-FABP from initiating PCTV budding. Upon the phosphorylation of SAR1B by PKCζ, the complex dissociates and L-FABP begins PCTV budding. Both CD36 and VAMP7 are important for PCTV budding initiation. □ TANGO1/TALI are implicated in the exit of the PCTV from the ER □ The COPII proteins SEC23/24, SEC13/31, and SAR1B, present on the PCTV, are not required for PCTV budding and ER exit but are important for docking at the Golgi. Abbreviations: SAR1B, secretion associated Ras related GTPase; SVIP, small valosin-containing protein-interaction protein; L-FABP, liver fatty acid-binding protein; PKCζ, protein kinase C zeta; CD36, cluster of differentiation 36; VAMP7, vesicle associated membrane protein 7; TALI, TANGO1-like protein; PCTV, prechylomicron transport vesicle. Figure made in BioRender.

The PCTVs are unique from other intracellular transport vesicles and several proteins needed for the formation of these vesicles have been described previously and are discussed below (summarized in Fig. 2).

The initiation of PCTV formation has been shown to depend on the liver fatty acid-binding protein (L-FABP) [43]; *Fabp1*^*-/-*^ mice fed a Western-type diet show triglycerides accumulation in intestinal cells, and a reduced chylomicron output from the intestine [44]. Siddiqi *et al.* suggest that L-FABP is initially inhibited from initiating PCTV formation by protein complex composed of SAR1B, small valosin-containing protein-interacting protein (SVIP), and SEC13. Upon SAR1B's phosphorylation by protein kinase C, zeta (PKCζ), the complex dissociates thereby freeing L-FABP and allowing it to bind to the ER [45]. Interestingly, loss of SAR1B does not prevent budding of PCTVs from the ER in rat enterocytes but these vesicles cannot fuse to the *cis*-Golgi [46]. SVIP, a regulator of VLDL trafficking [47], has thus far only been shown to be present in the above described complex in rat intestinal cytosol [45] but its role in PCTV budding/trafficking is yet to be described.

CD36 has also been shown to be a requirement for budding of the PCTV based on studies in mice, and mouse and rat enterocytes [39,48]. In addition, vesicle associated membrane protein 7 (VAMP7) is also essential in facilitating budding from the ER in rat enterocytes [39].

While proteins of the coat protein complex II (COPII) are well known to assist in VLDL budding and trafficking [49–51], these proteins are not a requirement for PCTV budding [46]. Siddiqi *et al.* showed that depleting SAR1B or SEC31 from the cytosol of rat enterocytes still resulted in the formation and budding of PCTVs [46]. On the other hand, the COPII proteins SEC13/31, SEC23/24, and SAR1B are shown to be present on the PCTV of 150–500 nm [46]. Zanetti *et al.* have shown that in yeast, COPII proteins can arrange themselves into inner and outer COPII layers around artificial large circular and tubular membranes [52], supporting the notion that COPII proteins are able to rearrange themselves around vesicles larger than 100 nm, despite previous uncertainty on whether the COPII protein coat constrain vesicles to sizes smaller than 100 nm [51,53].

Another protein associated with PCTVs is SURF4. In murine liver, SURF4 is recruited to ER exit sites by SAR1B, where it functions as a cargo receptor for apoB-containing lipoproteins [54]. Loss of SURF4 in the murine small intestine results in severe lipid accumulation in enterocytes, along with a reduction in serum apoB48 [55^▪^] implying that SURF4 has a similar function in the small intestine as it does in the liver.

What occurs after the PCTV buds from the ER and how it is trafficked to the Golgi remains unclear. Santos *et al.* suggest a mechanism: TANGO1 and TANGO1-like protein (TALI) work together to export the PCTV to the ER-Golgi intermediate compartment (ERGIC) [44]. However, loss of LMAN1 (also known as ERGIC-53), a resident ERGIC protein known to play a role in ER to Golgi transport of selective cargo, does not present a lipid phenotype in mice, but rather a deficiency of factor V and/or VIII [56]. After exit from the ER, it has been suggested that PCTVs travel to the Golgi in LMAN1-free ERGIC tubules [57], but it is not known which proteins are involved [58].

## AT THE GOLGI, AND SECRETION

**FIGURE 3:**
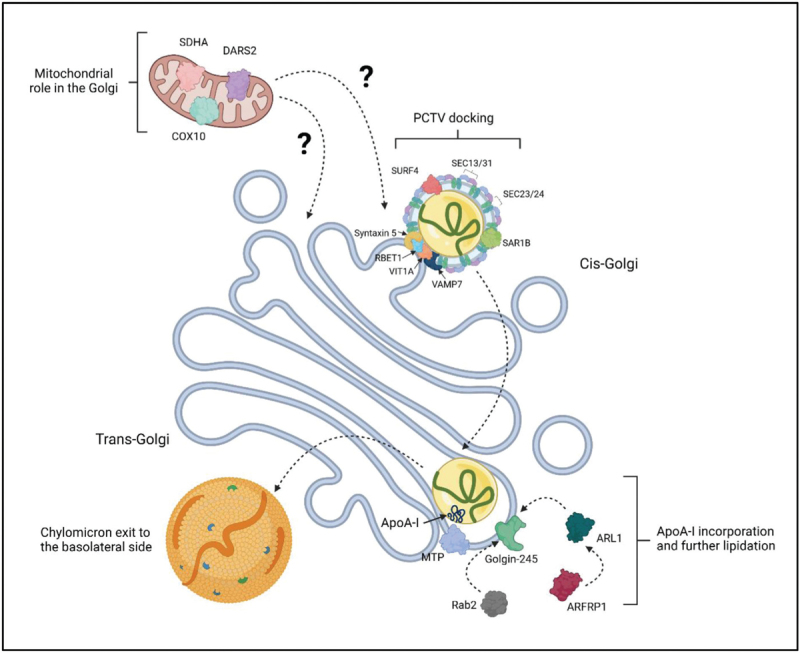
Docking of the prechylomicron transport vesicle (PCTV) at the Golgi apparatus. Intestinal loss of mitochondrial proteins DARS2, SDHA, and COX10 results in lipid accumulation and impaired chylomicron biosynthesis by disruption of the Golgi for unknown reasons. The COPII proteins, SEC23/24 are required for PCTV docking at the *cis*-Golgi. Additionally, VAMP7 and the t-SNARES Syntaxin-5, RBET1, and VIT1A are required for PCTV docking. MTP is present in the Golgi complex and could participate in further lipidation of chylomicrons. ARFRP1 and its downstream proteins ARL1, Golgin-245, and Rab2, are thought to aid in further lipidation of the chylomicron. The same ARL1-Golgin-245-Rab2 cascade is also responsible for the procurement of ApoAI. ApoB48 is further glycosylated in the Golgi. Abbreviations: VAMP7, vesicle associated membrane protein 7; VIT1A, Vps10p tail interactor-1a; MTP, microsomal triglyceride transfer protein; ARFRP1, ADP-ribosylation factor-related protein 1; ApoAI, apolipoprotein AI; ApoB48, apolipoprotein B48; DARS2, aspartyl-tRNA synthetase; SDHA, succinate dehydrogenase A; COX10, protohaem IX farnesyltransferase. Figure made in BioRender.

While the COPII complex is not required for PCTV budding, several of its proteins are needed for successful docking of the PCTV at the cis-Golgi (Fig. 3). For example, depletion of SEC23 and SEC24C in the cytosol of rat enterocytes cause a 25% reduction or complete loss of docking capacity, respectively [59]. Additionally, VAMP7, along with the t-SNARES, syntaxin 5, Vps10p-tail-interactor-1a (VIT1A), and RBET1, are also necessary for this process in this model, however, their precise mechanism remains unknown [60]. What occurs following docking at the Golgi is the maturation of chylomicrons which involves apoAI acquisition, apoB48 glycosylation, and further lipidation [1,7,61].

Functional MTP has been observed in the Golgi coupled with apoB in rat small intestine, implying further chylomicron lipidation [62,63] but direct evidence for this missing. The GTPase ADP-ribosylation factor-related protein 1 (ARFRP1) has also been proposed to be required for chylomicron maturation. This protein regulates the recruitment of ARL1 to the Golgi membrane where it binds to the scaffolding protein Golgin-245 which also binds Rab2. Interestingly, intestine-specific *Arfrp1*^*-/-*^ mice have no defects in MTP activity, but present with growth retardation, reduced fat and lean mass, and lipid-poor chylomicron secretion into the lymph [64]. Similarly, depletion of ARFRP1, ARL1, Golgin-245, and Rab2 in Caco-2 cells shows impaired secretion of triglycerides [64]. Second, the ARFRP1-ARL1-Golgin-Rab2 cascade is also suggested to play a role in the acquisition of apoAI to chylomicrons [64] which is dependent on SURF4 [33]. Finally, apoB48 is further glycosylated within the Golgi [65,66].

Intriguingly, mitochondrial proteins, aspartyl-tRNA synthetase (DARS2), succinate dehydrogenase A (SDHA), and protohaem IX farnesyltransferase (COX10), have recently been identified as new players in intracellular chylomicron metabolism. Loss in either of these proteins results in lipid accumulation in mouse enterocytes [67^▪^]. In intestinal *Dars2*^*-/-*^ mice, disruption of the Golgi results in impaired chylomicron production and/or PCTV trafficking from the ER to the Golgi associated with failure to thrive and a limited life span [67^▪^]. The study suggests that the accumulating lipids are of dietary origin. The intestinal ablation of specific OXPHOS subunits (SDHA or COX1) in mice displays similar phenotypes [67^▪^]. These findings imply that normal mitochondrial function is required for chylomicron biosynthesis which warrants further study, especially when considering the discovery that mitochondria are also important in hepatic VLDL biosynthesis [68,69].

There is very little known about chylomicron budding from the Golgi and the subsequent trafficking to the basolateral side of enterocytes. This includes the range in diameter of the chylomicrons exiting the Golgi. DENND5B has been associated with chylomicron Golgi-to-plasma membrane transport in mice; *Denn5b*^*-/-*^ mice display enlarged, opaque small intestines, accumulate ‘chylomicron secretory vesicles’ after the Golgi, and exhibit reduced triglyceride secretion into the circulation [70]. However, the mechanism for this remains to be elucidated. Finally, how chylomicrons are eventually secreted out into the lymph remains unknown. For the transport of chylomicrons from the lymph to the circulation we refer to these reviews: [71–73].

## CONCLUSION

To conclude, TM6SF2, PLA2G12B, PRAP1, and SURF4, have recently been identified to play a role in chylomicron biosynthesis specifically at the ER. *In vitro* studies have provided little new information on the budding and subsequent docking of the PCTV to the *cis*-Golgi while *in vivo* relevance to substantiate these findings are often missing. Interestingly, mitochondrial dysfunction in the small intestine associated with dispersal of the Golgi apparatus was shown to impair trafficking of chylomicrons from the ER to the Golgi. What occurs at the Golgi and after remains largely a mystery. Thus, we believe that future insights into chylomicron biosynthesis require the efforts of a diverse range of experts to work together in identifying novel proteins, their functions, and elucidating intracellular mechanisms, to complete our understanding of chylomicron biosynthesis, trafficking, and secretion.

Finally, we wish to highlight the following outstanding questions:

(1) How can the enterocyte sustain the transport, maturation and exit of chylomicron particles up to 1200 nm in diameter?
(2) What are the requirements for vesicular exit of chylomicrons from the Golgi?
(3) How do chylomicrons exit the basolateral membrane into the lymph?

## Acknowledgements

*None.*

### Financial support and sponsorship

*None.*

### Conflicts of interest

*There are no conflicts of interest.*

## REFERENCES AND RECOMMENDED READING

Papers of particular interest, published within the annual period of review, have been highlighted as:

▪ of special interest
▪▪ of outstanding interest
